# Electronic Structure Modulation in MoO_2_/MoP Heterostructure to Induce Fast Electronic/Ionic Diffusion Kinetics for Lithium Storage

**DOI:** 10.1002/advs.202104504

**Published:** 2022-01-09

**Authors:** Yuanhao Shen, Yalong Jiang, Zhongzhuo Yang, Jun Dong, Wei Yang, Qinyou An, Liqiang Mai

**Affiliations:** ^1^ State Key Laboratory of Advanced Technology for Materials Synthesis and Processing Wuhan University of Technology Wuhan 430070 P. R. China; ^2^ Hubei Engineering Research Center for Safety Monitoring of New Energy and Power Grid Equipment Hubei University of Technology Wuhan 430068 P. R. China; ^3^ Foshan Xianhu Laboratory of the Advanced Energy Science and Technology Guangdong Laboratory Xianhu hydrogen Valley Foshan 528200 China

**Keywords:** heterosturctures, lithium‐ion batteries, long‐term cycling stability, MoO_2_/MoP

## Abstract

Transition metal oxides (TMOs) are considered as the prospective anode materials in lithium‐ion batteries (LIBs). Nevertheless, the disadvantages, including large volume variation and poor electrical conductivity, obstruct these materials to meet the needs of practical application. Well‐designed mesoporous nanostructures and electronic structure modulation can enhance the electron/Li‐ions diffusion kinetics. Herein, a unique mesoporous molybdenum dioxide/molybdenum phosphide heterostructure nanobelts (meso‐MoO_2_/MoP‐NBs) composed of uniform nanoparticles is obtained by one‐step phosphorization process. The Mott–Schottky tests and density functional theory calculations demonstrated that meso‐MoO_2_/MoP‐NBs possesses superior electronic conductivity. The detailed lithium storage mechanism (solid solution reaction for MoP and partial conversion for MoO_2_), small change ratio of crystal structure and fast electronic/ionic diffusion behavior of meso‐MoO_2_/MoP‐NBs are systematically investigated by operando X‐ray diffraction, ex situ transmission electron microscopy, and kinetic analysis. Benefiting from the synergistic effects, the meso‐MoO_2_/MoP‐NBs displays a remarkable cycling performance (515 mAh g^−1^ after 1000 cycles at 1 A g^−1^) and excellent rate capability (291 mAh g^−1^ at 8 A g^−1^). These findings can shed light on the behavior of the electron/ion regulation in heterostructures and provide a potential route to develop high‐performance lithium‐ion storage materials.

## Graphical abstract

The mesoporous molybdenum dioxide/molybdenum phosphide heterostructure nanobelts (meso‐MoO_2_/MoP‐NBs) is composed of uniform nanoparticles, which offers continuous electron/ion transport pathways. The obvious charge transfer from MoO_2_ to MoP and stable crystal structure during the lithiation/delithiation process lead to an excellent rate capability and long‐term cycling performance (515 mAh g^−1^ after 1000 cycles at 1 A g^−1^) for meso‐MoO_2_/MoP‐NBs.

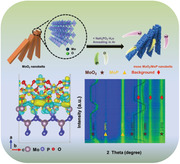

## 1 Introduction

Nowadays, lithium‐ion batteries (LIBs) are widely employed in various types of electronic equipment and electric vehicles on account of their environmental friendliness, prolonged cycling life and high energy density.^[^ ^1^ ^]^ Graphite has been regarded as anode electrode material for commercialization. Nevertheless, on account of its low theoretical capacity (≈372 mAh g^−1^) and volume capacity (≈850 mAh cm^−3^), graphite cannot meet the growing requirements of energy density.^[^ ^2^ ^]^ Consequently, there is an urgent need to explore a suitable anode material substitute. Compared with graphite, transition metal oxides (TMOs) have drawn much attention because of their high theoretical capacity.^[^ ^3^ ^]^ Among them, molybdenum dioxide (MoO_2_) is a promising candidate owing to its high chemical stability, low price, nontoxicity, high density (6.5 g cm^−3^), and high theoretical capacity (838 mAh g^−1^).^[^ ^4^ ^]^ In addition, MoO_2_ has been regarded as a relatively secure anode material because the Li insertion voltage of MoO_2_ is higher than that of commercial graphite.^[^ ^5^ ^]^ However, the disadvantages of MoO_2_ inhibit its practical applications, such as its inherent low electronic conductivity and the rapid destruction of the material structure caused by the large and irreversible volume expansion during Li^+^ insertion/extraction process, further resulting in inferior reaction kinetics and cycling stability.^[^ ^5^, ^6^ ^]^

Well‐designed mesoporous nanostructures could increase the effective contact areas between the electrolytes and electrode materials, meanwhile, accommodate the volume changes, further improving the Li^+^ diffusion kinetics and storage performance.^[^ ^7^ ^]^ MoO_2_ has been synthesized by researchers into various nanosized morphology, such as nanoparticles,^[^ ^8^ ^]^ nanowires,^[^ ^9^ ^]^ nanobelts,^[^ ^10^ ^]^ nanorods,^[^ ^11^ ^]^ and nanosheets.^[^ ^12^ ^]^ Lou et al. prepared a triple‐shelled MoO_2_/carbon composite hollow spheres by high‐temperature calculation from Molybdenum‐Polydopamine hollow spheres precursor, which delivered an outstanding cycling performance (580 mAh g^−1^ at 0.5 A g^−1^ after 200 cycles).^[^ ^13^ ^]^ Nagaraju G et al. reported bare MoO_2_ nanoparticles displayed almost 3 times the capacity of commercial MoO_2_.^[8a]^ Among the different forms of nanomaterials, 1D mesoporous nanomaterials could offer many advantages for enhancing the charge storage performance: 1) high surface areas which ensure effective contact of the electrolyte and the electrode interface; 2) empty spaces which accommodate the volume changes; 3) short electron/ion transmission distance.^[^ ^14^ ^]^ Thus, it is effective to synthesize 1D mesoporous MoO_2_ nanomaterials to improve the lithium storage performance. However, the poor intrinsic electronic conductivity of MoO_2_ remains unsolved.

Up to now, the intrinsic poor conductivity of electrode materials could be improved by electronic structure modulation, which usually contains three ways: introducing dopants,^[^ ^15^ ^]^ manufacturing defects,^[^ ^16^ ^]^ and constructing heterostructures.^[1a,17]^ Among them, heterostructures usually promote the separation of electrons and holes in both materials, thus resulting in higher electronic mobility than that of pure materials.^[^ ^18^ ^]^ For instance, Chen et al. reported Mo_2_N nanolayer coated MoO_2_ hollow heterogeneous nanostructures, delivering excellent specific capacity up to 815 mAh g^−1^, long‐term cycling stability and superior rate capability.^[^ ^5^ ^]^ Dou et al. proposed Fe_3_O_4_/FeS heterostructures by coprecipitation and subsequent partial sulfurization to induce a highly active and stable electrode structure, delivering an outstanding cycling performance (913.9 mAh g^−1^ after 1000 cycles at 1 A g^−1^) and high rate capability (490.4 mAh g^−1^ at 10 A g^−1^).^[^ ^19^ ^]^ These unique heterostructures display tunable electronic properties, resulting in improved dynamics and structural stability. Furthermore, molybdenum phosphide (MoP) exhibits moderate operation potential, high electronic conductivity (>5000 S cm^−1^) and superior electrochemical activity, which delivers promising lithium‐ion storage performance.^[^ ^20^ ^]^ Above all, constructing 1D mesoporous MoO_2_/MoP heterostructure nanomaterials could be a promising approach to improve lithium‐ion storage performance.

**Figure 1:**
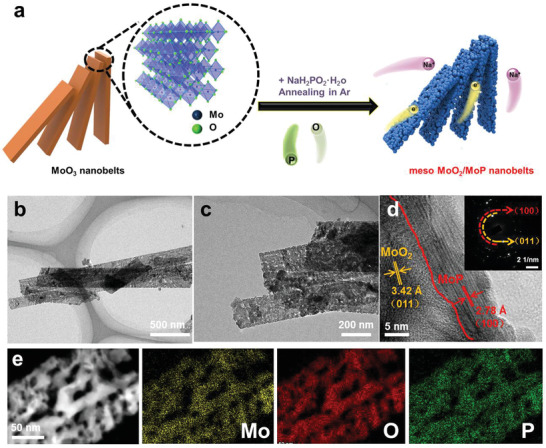
Synthesis route and morphology characterizations of meso‐MoO_2_/MoP‐NBs. a) Schematic of synthesis process. b,c) Bright field TEM images. d) HRTEM image and inset is the related SAED patterns. e) HAADF‐STEM image and EDX mapping images.

Herein, we synthesized meso‐MoO_2_/MoP‐NBs composed of uniform nanoparticles by a facile high‐temperature phosphating treatment, which offers continuous electron/ion transport pathways (**Figure** **1**a). Meanwhile, the detailed lithium‐ion storage mechanism and the relationship between the two components in heterostructure were investigated by systematic operando X‐ray diffraction (XRD), ex situ transmission electron microscopy (TEM), density functional theory (DFT) calculations and kinetics analysis. The meso‐MoO_2_/MoP‐NBs exhibits more electron states and smaller changes in crystal structure parameters during lithium‐ion intercalation/extraction, indicating high electron conductivity and excellent structural stability. It is of novelty to design the heterostructure materials consisting of two parts with different types of electronic structure to achieve superior electronic conductivity. Therefore, the rational designed meso‐MoO_2_/MoP‐NBs exhibits enhanced electrochemical performance.

## 2 Results and Discussion

Layered *α*‐MoO_3_ nanobelts were prepared by hydrothermal method.^[^ ^21^ ^]^ It is highly crystalline with 15–20 µm in length and a width of about 200 nm (Figure S1, Supporting Information). The precursor *α*‐MoO_3_ is easier to be reduced because of its high‐valence molybdenum element. The occurrence of phosphating reaction accompanied with anions exchange process (phosphorus substitutes oxygen) converts *α*‐MoO_3_ into MoP.^[^ ^22^ ^]^ The degree of phosphating was controlled by regulating the pressure in the quartz tube. In other words, the meso‐MoO_2_‐NBs, meso‐MoO_2_/MoP‐NBs, and meso‐MoP‐NBs would be prepared when gradually increasing the mass of NaH_2_PO_2_·H_2_O.

The morphology characteristics were evaluated by scanning electron microscopy (SEM) for all three samples. The SEM images of meso‐MoO_2_/MoP‐NBs (Figure S2a, Supporting Information), meso‐MoP‐NBs (Figure S2b, Supporting Information), and meso‐MoO_2_‐NBs (Figure S2c, Supporting Information) show the uniform nanobelts morphology with a width of about 150–200 nm. To obtain the microstructure of as‐prepared samples, the mesoporous nanobelt structures composed of uniform nanoparticles were under TEM observation (Figure 1b,c; and Figures S3a,b and S4a,b, Supporting Information). Moreover, the high‐resolution TEM (HRTEM) image of meso‐MoO_2_/MoP‐NBs displays the lattice fringes of about 3.42 and 2.78 Å, which, respectively, correspond to the characteristic (011) plane of MoO_2_ and (100) plane of MoP (Figure 1d). As indicated by the red line, the phase boundary between MoO_2_ and MoP could be observed clearly. The selected area electron diffraction (SAED) pattern reveals the polycrystalline feature of meso‐MoO_2_/MoP‐NBs (inset of Figure 1d). The high‐angle annular dark field‐scanning transmission electron microscopy (HAADF‐STEM) (Figure 1e) and the relevant energy‐dispersive X‐ray (EDX) mapping confirm the equal distribution of Mo, P, and O elements in meso‐MoO_2_/MoP‐NBs. Additionally, HRTEM images of meso‐MoP‐NBs (Figure S3c, Supporting Information) reveal the fringe spacings of 2.79 Å belonging to the (100) plane of MoP. Meanwhile, the fringe spacing of meso‐MoO_2_‐NBs is 2.15 Å which agrees well with the (210) plane of MoO_2_ (Figure S4c, Supporting Information). The Mo, P, and O elements homogeneously distribute in meso‐MoP‐NBs (Figure S3d, Supporting Information) and meso‐MoO_2_‐NBs (Figure S4d, Supporting Information), as confirmed by HAADF‐STEM and the corresponding mapping images, which match well with the inductively coupled plasma (ICP) data (Table S2, Supporting Information). Interestingly, meso‐MoO_2_‐NBs contains the P element, while meso‐MoP‐NBs contains the O element. The P element exists in meso‐MoO_2_‐NBs because of the partial P residue in the phosphating process. As for meso‐MoP‐NBs, when exposed to air, slight oxidation usually occurs on the surface because of its high surface energy.^[^ ^23^ ^]^

**Figure 2:**
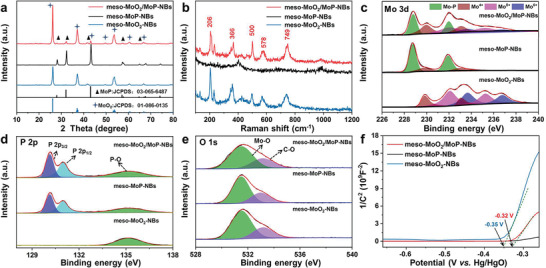
Phase characterizations of meso‐MoO_2_/MoP‐NBs, meso‐MoP‐NBs, and meso‐MoO_2_‐NBs, respectively. a) XRD patterns. b) Raman spectra. c–e) High‐resolution XPS scans of Mo 3d, P 2p, and O 1s. f) Mott–Schottky analysis.

The XRD patterns of as‐prepared samples are displayed in **Figure** **2**a. The main diffraction peaks are attached to monoclinic MoO_2_ (JCPDS: 01‐086‐0135, space group: *P*21/c) and hexagonal MoP (JCPDS: 03‐065‐6487, space group: *P*‐6m2). During different degrees of phosphating treatment, the *α*‐MoO_3_ nanobelts are reductively converted into MoO_2_, the mixture of MoO_2_ and MoP, and MoP. The feature peaks of monoclinic MoO_2_ located at 26.01°, 41.86°, and 49.55° correspond to the crystal faces (011), (−122), and (‐212), respectively. Besides, the broad peaks at 36.99° and 53.49° refer to a combination of multiple diffraction peaks. The main peaks of hexagonal MoP are observed at 27.94°, 32.04°, 43.02°, and 57.11°, corresponding to the crystal faces (001), (100), (101), and (110). Raman spectra between 100 and 1200 cm^−1^ were recorded to further study the properties of as‐prepared samples (Figure 2b). The peaks observed at 206, 366, 500, 578, 749 cm^−1^ are assigned to the monoclinic MoO_2_.^[^ ^24^ ^]^ Besides, the two main peaks at 403 and 983 cm^−1^ are attached to MoP.^[^ ^25^ ^]^ X­‐ray photoelectron spectroscopy (XPS) measurements were applied to analyze surface chemical compositions of as‐prepared samples. The survey spectra show that the Mo, O, C, and P elements all exist in meso‐MoO_2_/MoP‐NBs, meso‐MoP‐NBs, and meso‐MoO_2_‐NBs samples (Figure S5a, Supporting Information), which match well with EDX mapping and ICP data. Figure 2c displays the Mo 3d region. One doublet situated at 231.9 and 228.8 eV are ascribed to MoP.^[^ ^23^, ^26^ ^]^ Besides, the other two pairs of peaks at 229.9/233.2 eV (Mo 3d_5/2_/Mo 3d_3/2_) and 233.7/236.8 eV (Mo 3d_5/2_/Mo 3d_3/2_) are assigned to high oxidation states of Mo (Mo^4+^ and Mo^6+^),^[^ ^23^, ^27^ ^]^ related to the high‐valence molybdenum oxides. As reported by Dunn et al., a pair of peaks located at 230.5 and 233.6 eV refer to Mo^5+^.^[^ ^28^ ^]^ Therefore, the peaks of 232.1 and 235.3 eV are attributed to Mo^*δ*+^, because they are located between the positions of Mo^5+^ and Mo^6+^. Moreover, the fitting results of Mo 3d spectra indicate that molybdenum oxides with different valence states (Mo^4+^, Mo^*δ*+^, and Mo^6+^) exist in meso‐MoO_2_‐NBs (Figure S5b, Supporting Information). After the phosphating treatment, molybdenum oxides are gradually reduced to low‐valence molybdenum compounds. When fully reduced to MoP, there still exist Mo^4+^ in meso‐MoP‐NBs, resulting from the slight oxidation on the surface of MoP in air.^[^ ^23^ ^]^ Figure 2d displays the P 2p spectra of three samples. The doublet at 130.13 and 131.03 eV in meso‐MoO_2_/MoP‐NBs and meso‐MoP‐NBs are associated with P 2p_3/2_ and P 2p_1/2_ of MoP.^[^ ^26^ ^]^ Simultaneously, the P—O bond is located at 135.23 eV of all samples. The P—O bond exists in meso‐MoO_2_‐NBs could be attributed to PO_4_^3−^ due to superficial oxidation in air.^[^ ^29^ ^]^ The O 1s spectra reveal that the Mo—O bond is located at 531.5 eV and the C—O bond is centered at 533.3 eV, respectively (Figure 2e).^[^ ^30^ ^]^ Mott–Schottky (M–S) curves were tested to explain the MoO_2_/MoP heterostructure in Figure 2f. The flat band potential (*E*_FB_) of meso‐MoP‐NBs is tough to obtain due to its high conductivity. Meanwhile, obvious positive slopes for meso‐MoO_2_/MoP‐NBs and meso‐MoO_2_‐NBs could be observed. Moreover, the *E*_FB_ of three samples could be based on the following Equation (1)^[^ ^31^ ^]^

(1) $$\frac{1}{C^{2}}=\frac{2}{A^{2}e\varepsilon \varepsilon_{0}N_{A}}\;\left(E-E_{\mathrm{FB}}-\frac{k_{B}T}{e}\right)$$

where, *C* and *E* represent the interfacial capacitance and applied potential, respectively. The constant *A* and *ε*_0_ are area and vacuum permittivity. Besides, *ε* is the dielectric constant of the semiconductor, *T* is the absolute temperature, *k*_B_ is the Boltzmann constant and *e* is the electronic charge.^[17c]^ The *E*_FB_ of meso‐MoO_2_/MoP‐NBs (−0.32 V vs Hg/HgO) is higher than that of meso‐MoO_2_‐NBs (−0.35 V vs Hg/HgO), suggesting favorite migration of electrons from MoO_2_ to MoP. The nitrogen adsorption isotherm curves of the meso‐MoO_2_/MoP‐NBs, meso‐MoP‐NBs, and meso‐MoO_2_‐NBs are shown in Figure S6a (Supporting Information). The corresponding specific surface areas are 363.1, 628.3, and 418.7 m^2^ g^−1^. Meanwhile, the pore size (meso‐MoO_2_/MoP‐NBs: 4.4 nm; meso‐MoP‐NBs: 3.7 nm; meso‐MoO_2_‐NBs: 4.5 nm) further confirms the unique mesoporous structure (Figure S6b, Supporting Information).

**Figure 3:**
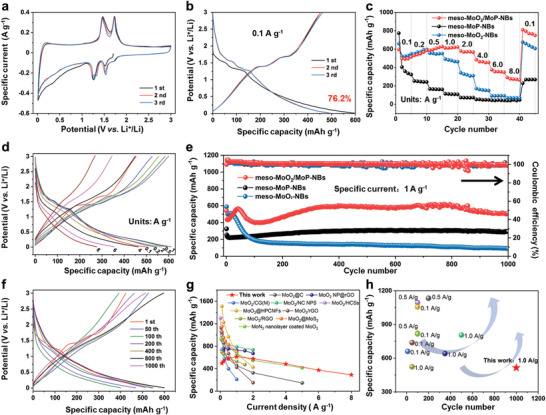
Lithium‐ion storage performance. a) CV curves of meso‐MoO_2_/MoP‐NBs at the first three cycles, with a sweep rate of 0.2 mV s^−1^. b) Galvanostatic charge–discharge curves at 0.1 A g^−1^ of meso‐MoO_2_/MoP‐NBs. c) Rate performance of meso‐MoO_2_/MoP‐NBs, meso‐MoP‐NBs, and meso‐MoO_2_‐NBs. d) Galvanostatic charge–discharge curves of meso‐MoO_2_/MoP‐NBs at different current densities in the range of 0.1 and 8 A g^−1^.e) Long‐term cycling performance at 1 A g^−1^ of meso‐MoO_2_/MoP‐NBs, meso‐MoP‐NBs, and meso‐MoO_2_‐NBs, respectively. f) Galvanostatic charge–discharge curves of meso‐MoO_2_/MoP‐NBs at different cycles at 1 A g^−1^. g,h) Rate performance and cycling performance of this work compared with the reported MoO_2_‐based anode materials for LIBs (The detail was listed in Table S4, Supporting Information).

The electrochemical behavior of three samples was assessed by the assembly of half‐cells (2016‐type). As shown in the cyclic voltammetry (CV) curves, the meso‐MoO_2_/MoP‐NBs electrode was tested at the scan rate of 0.2 mV s^−1^ in 0.01–3 V versus Li^+^/Li during the initial three cycles (**Figure** **3**a). Two couples of reversible redox pairs at 1.52/1.73 and 1.25/1.46 V versus Li^+^/Li are associated with the reversible phase transformed process of Li*_x_* MoO_2_ between monoclinic and orthorhombic phase, as described in Equation (2).^[^ ^32^ ^]^ When the voltage is lower than 1 V versus Li^+^/Li, metallic Mo and Li_2_O are obtained by conversion reaction (Equation (3)).^[^ ^33^ ^]^ The overlapped CV curves of two subsequent cycles indicate the highly reversible lithium‐ion storage behavior. In addition, a redox pair of peaks observed at 0.93/1.51 V versus Li^+^/Li corresponds to the reversible solid solution reaction for MoP, as displayed in Equation (4) (Figure S9a, Supporting Information)^[^ ^34^ ^]^

(2) $$\mathrm{Mo}O_{2}+xLi^{+}+xe^{-}↔Li_{x}\mathrm{Mo}O_{2}\left(0\leq x\leq 0.98\right)\;$$

(3) $${\mathrm{Li}}_{x}\mathrm{Mo}O_{2}+\left(4-x\right){\mathrm{Li}}^{+}+\left(4-x\right)e^{-}↔2{\mathrm{Li}}_{2}O+\mathrm{Mo}$$

(4) $$\mathrm{MoP}+xLi^{+}+xe^{-}↔Li_{x}\mathrm{MoP}\;$$

The charge–discharge curves of meso‐MoO_2_/MoP‐NBs show that the initial discharge/charge capacities are 596/454 mAh g^−1^ (coulombic efficiency of 76.2%) (Figure 3b). Meanwhile, the initial coulombic efficiency of meso‐MoO_2_‐NBs and meso‐MoP‐NBs is 73.5% and 47.3%, which is lower than meso‐MoO_2_/MoP‐NBs (Figure S7a,b, Supporting Information). This is because the specific surface areas of meso‐MoO_2_/MoP‐NBs is the lowest among the three samples (Figure S6a, Supporting Information). The larger contact area of the electrode material with the electrolyte means that more lithium ions need to be consumed to form the SEI (Figure S8, Supporting Information).^[8a]^ There are two reversible discharge/charge plateaus at about 1.68/1.42 and 1.32/1.71 V versus Li^+^/Li for meso‐MoO_2_/MoP‐NBs, which match well with CV results. Figure 3c reveals a remarkable rate performance of meso‐MoO_2_/MoP‐NBs. At 0.1 A g^−1^, meso‐MoO_2_/MoP‐NBs could reach to 496 mAh g^−1^. When the current density reaches up to 8 A g^−1^, meso‐MoO_2_/MoP‐NBs displays a reversible capacity of 291 mAh g^−1^. Compared with meso‐MoO_2_‐NBs and meso‐MoP‐NBs, the meso‐MoO_2_/MoP‐NBs exhibits the most understanding rate performance. At various current densities within the scope of 0.1–8 A g^−1^, the relevant galvanostatic charge–discharge plots of meso‐MoO_2_/MoP‐NBs indicate that the polarization is small as shown in Figure 3d. As for cycling performance, the as‐prepared three samples were tested at the current density of 1 A g^−1^ in Figure 3e. When cycling to 1000 cycles, the meso‐MoO_2_/MoP‐NBs delivers a highly reversible specific capacity of 515 mAh g^−1^, which is better than that of meso‐MoO_2_‐NBs (91 mAh g^−1^) and meso‐MoP‐NBs (292 mAh g^−1^). Figure 3f displays the typical galvanostatic charge–discharge curves of meso‐MoO_2_/MoP‐NBs electrode with different cycles (1st, 50th, 100th, 200th, 400th, 800th, 1000th) at 1 A g^−1^ in the voltage range of 0.01 and 3 V versus Li^+^/Li. Interestingly, the reversible capacity gradually increases in the first 50 cycles. This special activated process could be ascribed to that the intercalation of Li ions can weaken the interaction between Mo and O, and promote the formation of Mo vacancies, providing more reaction sites. Thus, it could enhance the lithium ions storage capacity.^[9b,35]^ To highlight the outstanding lithium storage performance of meso‐MoO_2_/MoP‐NBs, its position of cycling stability and rate performance within the reported MoO_2_‐based LIB electrodes were listed in Figure 3g,h. A more comprehensive data comparison was listed in Table S4 (Supporting Information). It is worth noting that when increased to the high current density of 8 A g^−1^, a superior capacity of 291 mAh g^−1^ (59% of the reversible 496 mAh g^−1^ at 0.1 A g^−1^) could be achieved, demonstrating its superior rate performance which is not commonly seen for MoO_2_‐based LIB electrodes, while also highlighting the significance of its practical application value.

**Figure 4:**
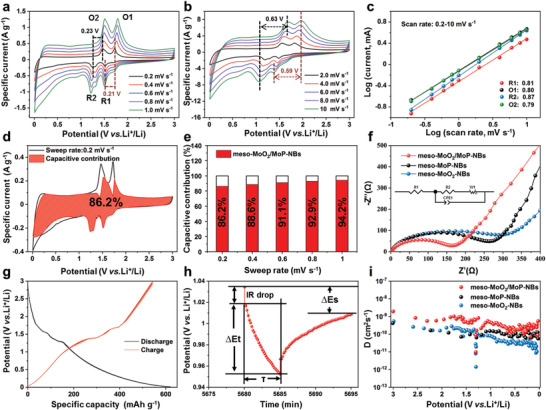
Kinetics analysis of lithium‐ion storage. a,b) CV curves of meso‐MoO_2_/MoP‐NBs at the sweep rates ranging from 0.2 to 10 mV s^−1^. c) Log (i) versus log (v) plots at different oxidation and reduction states. d) CV curve of meso‐MoO_2_/MoP‐NBs at 0.2 mV s^−1^, the hatched portion shows the capacitive controlled regions. e) The ratio of capacitive contribution at different scan rates of meso‐MoO_2_/MoP‐NBs. f) Nyquist plots of meso‐MoO_2_/MoP‐NBs, meso‐MoP‐NBs, and meso‐MoO_2_‐NBs. g) GITT curves of meso‐MoO_2_/MoP‐NBs. h) GITT potential response curve with time. The experiment was carried out at constant current pulse of 20 mA g^−1^ for 5 min followed by a relaxation period of 10 min. i) Diffusion coefficient at different discharge states.

The Li^+^ diffusion kinetics analysis was evaluated by the CV test. The CV curves of meso‐MoO_2_/MoP‐NBs, meso‐MoP‐NBs and meso‐MoO_2_‐NBs with diverse scan rates from 0.2 to 10 mV s^−1^ were shown in **Figure** **4**a,b, and Figures S9a,b and S10a,b (Supporting Information). The redox peaks of the meso‐MoO_2_/MoP‐NBs exhibit the smaller shift with increasing scan rates, indicating the lower polarization and faster reaction kinetics compared to meso‐MoO_2_‐NBs. The connection with peak current (*i*) and scan rate (*v*) could be calculated in Equation (5)^[^ ^36^ ^]^

(5) $$i\;=av^{b}$$

The *b* values could be obtained by fitting log (*i*) versus log (*v*). When the storage performance belongs to semi‐infinite diffusion‐controlled, the *b*‐value is close to 0.5, while the *b*‐value of nearly 1 means a capacitance‐dominated behavior.^[^ ^36^ ^]^ For meso‐MoO_2_/MoP‐NBs, the *b*‐value of two reversible redox pairs (R1/O1 and R2/O2) is 0.81/0.80 and 0.87/0.79 (Figure 4c), which are larger than that of 0.77/0.74 and 0.79/0.72 for meso‐MoO_2_‐NBs (Figure S10d, Supporting Information), suggesting that the meso‐MoO_2_/MoP‐NBs exhibits the faster reaction kinetics. Meanwhile, the meso‐MoP‐NBs with intrinsically high conductivity shows the highest *b*‐value among the three samples (Figure S9d, Supporting Information). Besides, Dunn and co­workers proposed a method to calculate total capacitive contribution proportion.^[^ ^37^ ^]^ The overall current *i*(*v*) could be divided into two parts: capacitive (*k*_1_ *v*) and diffusion controlled (*k*_2_ *v* ^1/2^) current responses, as calculated by following Equation (6)^[^ ^38^ ^]^

(6) $$i\left(v\right)=k_{1}v+k_{2}v^{1/2}$$

where *v* is the sweep rate. The capacitive contribution regions (shaded area) compared to the typical voltage profiles of meso‐MoO_2_/MoP‐NBs, meso‐MoP‐NBs, and meso‐MoO_2_‐NBs were measured at 0.2 mV s^−1^ in Figure 4d; and Figures S9c and S10c (Supporting Information). The capacitive contributions of meso‐MoO_2_/MoP‐NBs, meso‐MoP‐NBs, and meso‐MoO_2_‐NBs are 86.2%, 61.2%, and 76.1%, which indicate capacitive response plays a leading role in the charge storage process. Figure 4e exhibits that the capacitive contribution of meso‐MoO_2_/MoP‐NBs are 86.2%, 88.6%, 91.1%, 92.9%, and 94.2% at the scan rates of 0.2, 0.4, 0.6, 0.8, and 1.0 mV s^−1^, demonstrating the excellent rate capability. To further research the kinetics process, the electrochemical impedance spectrum (EIS) was deemed as an effective method (Figure 4f). The Nyquist plot includes the medium‐frequency region which corresponds to the charge transfer resistance (*R*_ct_) and the low‐frequency region represents the signal of ion diffusion resistance. The decreased *R*_ct_ of meso‐MoO_2_/MoP‐NBs (162.9 Ω), compared to meso‐MoO_2_‐NBs (306.7 Ω) and meso‐MoP‐NBs (267.2 Ω) suggests that meso‐MoO_2_/MoP‐NBs has a superior electron conductivity. Furthermore, based on the EIS data and following Equations (7) and (8)^[^ ^39^ ^]^

(7) $$D\;=\left(R^{2}T^{2}\right)/\left(2A^{2}n^{4}F^{4}C^{2}\sigma^{2}\right)$$

(8) $$Z^{\prime }=R_{D}\;+R_{L}+\sigma \omega^{-1/2}$$

where *D* is the lithium ion diffusion coefficient, *R* is the gas constant, *T* is the absolute temperature, *A* is the surface area of the anode, *n* is the number of electrons per molecule during reaction, *F* is the faraday constant, *C* is the concentration of lithium ion, the simulation of Warburg factor (*σ*) originated from the slope in fitting line of *ω* ^−1/2^ and *Z*′was carried out to evaluate the diffusion kinetics. As shown in Figure S11 (Supporting Information), the slope value of meso‐MoO_2_/MoP‐NBs (*σ*_1_ = 273) is smaller than that of meso‐MoP‐NBs (*σ*_3_ = 336) and meso‐MoO_2_‐NBs (*σ*_2_ = 451), indicating the best ion diffusion ability according to Equation (7). The Li^+^ solid‐state diffusion kinetics for meso‐MoO_2_/MoP‐NBs, meso‐MoP‐NBs, and meso‐MoO_2_‐NBs were calculated on galvanostatic intermittent titration technique (GITT) (Figure 4g; and Figure S12a,b, Supporting Information).^[^ ^40^ ^]^ Moreover, the Li^+^ diffusion coefficient ($D_{Li^{+}}$) could be obtained as the following formula^[^ ^41^ ^]^

(9) $$D_{Li^{+}}=\frac{4}{\pi \tau }\;{\left(\frac{m_{B}V_{M}}{M_{B}S}\right)}^{2}{\left(\frac{\Delta E_{s}}{\Delta E_{\tau }}\right)}^{2}$$

where *τ* is the constant current pulse duration, *m*_B_, *V*_M_, *S*, and *M*_B_ are the active mass, molar volume, surface area of electrode materials, and molar mass of meso‐MoO_2_/MoP‐NBs, respectively. Figure 4h displays the voltage difference (Δ*E*_s_) during the open circuit moment and the total change of cell voltage (Δ*E*_*τ*_) in the time of a constant current pulse without the *IR* drop. As shown in Figure 4i, the diffusion coefficient of meso‐MoO_2_/MoP‐NBs is ≈4×10^−10^ cm^2^ s^−1^, which is higher than that of meso‐MoO_2_‐NBs (1×10^−10^ cm^2^ s^−1^) and meso‐MoP‐NBs (6.5×10^−11^ cm^2^ s^−1^), indicating the enhanced Li^+^ diffusion kinetic, further resulting in excellent electrochemical performance.

**Figure 5:**
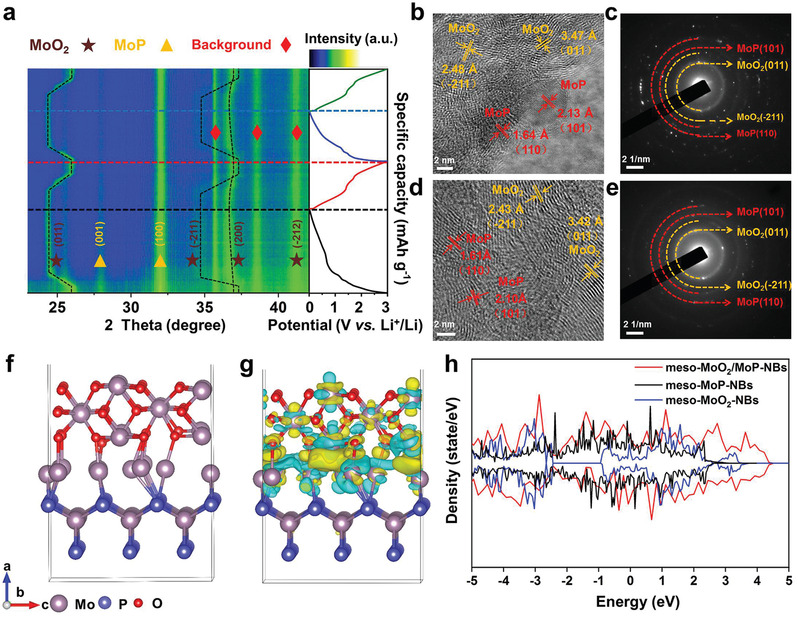
Lithium‐ion storage mechanism of meso‐MoO_2_/MoP‐NBs. a) Operando XRD patterns of meso‐MoO_2_/MoP‐NBs at the first three cycles. b) HRTEM image and c) SAED pattern of meso‐MoO_2_/MoP‐NBs at fully discharged state. d) HRTEM image and e) SAED pattern of meso‐MoO_2_/MoP‐NBs at fully charged state. f) Geometrically optimized model of MoP(100)/MoO_2_(011) heterojunction. g) Charge‐density difference of meso‐MoO_2_/MoP‐NBs. h) Calculated DOS of the meso‐MoO_2_/MoP‐NBs, meso‐MoP‐NBs, and meso‐MoO_2_‐NBs, respectively.

The lithium­ion storage mechanism of all samples was conducted by operando XRD and ex situ TEM. Operando XRD patterns of meso‐MoO_2_/MoP‐NBs (**Figure** **5**a), meso‐MoO_2_‐NBs (Figure S13a, Supporting Information) and meso‐MoP‐NBs (Figure S13b, Supporting Information) were performed during the galvanostatic test at 0.1 A g^−1^ in the first two cycles, respectively. Continuous lithiation in the process of discharging, the positions of MoO_2_ (011), MoO_2_ (‐211), and MoO_2_ (200) simultaneously transfer to lower 2*θ* position, which corresponds to the solid solution reaction of MoO_2_, and thus leads to expanded interlayer spacing. The shift of MoO_2_ (−212) peak is not obvious as a result that this peak overlaps with the background peak. The obvious offset peaks mentioned above remain stable at about 1 V versus Li^+^/Li, indicating partial conversion of lithiated MoO_2_ into metallic Mo and Li_2_O. This conclusion matches well with galvanostatic charge–discharge curves (Figure 3d). However, Mo metal and Li_2_O phase could not be detected by the operando XRD measurement, probably because of the nanosize dispersion.^[^ ^42^ ^]^ Besides, the peak locations of MoP remain unchanged during cycling, indicating that the limited solid‐solution reaction does not lead to the expansion of MoP crystal structure. The XRD patterns of operando testing device without electrodes are displayed in Figure S14 (Supporting Information). When discharged to 0.01 V versus Li^+^/Li, the HRTEM image of meso‐MoO_2_/MoP‐NBs displays the lattice fringes of about 3.47 and 2.48 Å, which correspond to the (011) and (−211) plane of MoO_2_ (Figure 5b), suggesting the larger lattice fringes compared to the initial state. The corresponding SAED pattern further confirms the polycrystallization nature of the meso‐MoO_2_/MoP‐NBs (Figure 5c). Besides, the fully delithiation state of meso‐MoO_2_/MoP‐NBs was also measured by HRTEM image and SAED pattern (Figure 5d,e). The spacings of the (011)/( −211) plane of MoO_2_ decrease to 3.42/2.43 Å and nearly return to the original state. While the spacings of the (101)/(110) plane of MoP remain unchanged during the lithiation/delithiation process. These results are in accordance with the operando XRD. To illustrate the structural stability of meso‐MoO_2_/MoP‐NBs, meso‐MoO_2_/MoP‐NBs maintains the nanobelts structure after 50 cycles (Figure S15a, Supporting Information). Besides, nanobelts structure and phase of meso‐MoO_2_/MoP‐NBs show no change even after 1000 cycles (Figure S15b,c, Supporting Information). Additionally, based on the relationship of a, b, c, h, k, l, and *β* in the monoclinic system, the lattice parameters include a, b, c, and cell volume (*V)* could be calculated. The lattice parameter could be obtained from (011), (−211), (200), and (−212) planes of MoO_2_ by Equation (10).^[^ ^43^ ^]^ As for meso‐MoO_2_‐NBs, when discharged to 0.01 V versus Li^+^/Li, the a, b, c, and *V* are 5.66, 4.94, 5.96, and 166.59 Å^3^. Compared to the initial state, the ratio of change about a, b, c, and *V* is 0.86%, 1.73%, 5.92%, and 8.68%. While for meso‐MoO_2_/MoP‐NBs, the rate of change of a, b, c and *V* are 0.32%, 1.57%, 5.82%, and 7.83%, which are smaller than that of meso‐MoO_2_‐NBs during cycling (Table S3, Supporting Information)

(10) $$\frac{1}{d^{2}}=\frac{h^{2}}{a^{2}\sin^{2}\beta }\;+\frac{k^{2}}{b^{2}}+\frac{l^{2}}{c^{2}\sin^{2}\beta }-\frac{2lh\cos\beta }{ca\sin^{2}\beta }$$

To obtain fundamental insight into the effect of heterostructures on electronic conductivity and structure stability, DFT calculations were conducted. The (100) plane of MoP and (011) plane of MoO_2_ are selected to construct the MoO_2_/MoP heterostructure, and the optimized model diagram is presented in Figure 5f. The obvious charge transfer that occurs from MoO_2_ to MoP in MoO_2_/MoP heterostructure can be shown in the charge density difference plot, which matches well with Mott–Schottky test results, thus leading to superior rate capability (Figure 5g). Further, the density of states (DOS) of meso‐MoO_2_/MoP‐NBs, meso‐MoP‐NBs and meso‐MoO_2_‐NBs are applied to analyze the charge transport (Figure 5h). Compared to meso‐MoP‐NBs and meso‐MoO_2_‐NBs, the additional continuous states from meso‐MoO_2_/MoP‐NBs are observed, which lead to a higher dispersion of band structure in meso‐MoO_2_/MoP‐NBs and enhance electronic conductivity. Partial density of states of meso‐MoO_2_/MoP‐NBs, meso‐MoP‐NBs, and meso‐MoO_2_‐NBs are displayed in Figure S16 (Supporting Information).

## 3 Conclusion

In summary, a new‐type mesoporous MoO_2_/MoP heterostructure nanobelts composed of uniform nanoparticles is successfully synthesized using a simple one‐step phosphorization method. The heterostructure promotes the separation of electrons and holes in both kinds of materials and the favorite migration of electrons from MoO_2_ to MoP, resulting in superior electronic conductivity, as demonstrated by Mott–Schottky curve tests and DFT calculations. When applied in LIBs, the meso‐MoO_2_/MoP‐NBs shows superior rate capability (291 mAh g^−1^ at 8 A g^−1^) and attractive cycling life. Pseudocapacitive mechanism of meso‐MoO_2_/MoP‐NBs with a high capacitive contribution up to 86.2% at 0.2 mV s^−1^ was revealed by electrochemical kinetic analysis. Operando XRD characterization and ex situ HRTEM tests clearly demonstrated that the lithium storage mechanism is the partial conversion of lithiated MoO_2_ and the limited solid‐solution reaction of MoP. The change ratio of a, b, c, and *V* for meso‐MoO_2_/MoP‐NBs is smaller than that of meso‐MoO_2_‐NBs during the lithiation/delithiation, benefiting for long‐term cycling stability. Our work presents MoO_2_/MoP heterostructure with high Li‐ion storage performance by electronic structure modulation. We are confident that the in‐depth insights into electronic structure and the detailed reaction mechanism could provide a new design criterion for advanced electrode materials in broad energy storage fields.

## Conflict of Interest

The authors declare no conflict of interest.

## Supporting information

Supporting Information

## Data Availability

The data that support the findings of this study are available from the corresponding author upon reasonable request.
