# Supplementary material for: Electronic Structure Modulation in MoO2/MoP Heterostructure to Induce Fast Electronic/Ionic Diffusion Kinetics for Lithium Storage
Source: Adv Sci (Weinh). 2022 Jan 9;9(6):2104504. doi: 10.1002/advs.202104504 (PMC8867142; doi:10.1002/advs.202104504)
Supplement: Supplementary file 1 — Supporting Information [file ADVS-9-2104504-s001.pdf]

ADVANCED SCIENCE
Open Access

Supporting Information

for *Adv. Sci.*, DOI: 10.1002/advs.202104504

Electronic Structure Modulation in MoO_2/MoP Heterostructure to Induce Fast Electronic/Ionic Diffusion Kinetics for Lithium Storage

Yuanhao Shen, Yalong Jiang, Zhongzhuo Yang, Jun Dong, Wei Yang, Qinyou An, and Liqiang Mai**

Supporting Information

Electronic Structure Modulation in MoO_2/MoP Heterostructure to Induce Fast Electronic/Ionic Diffusion Kinetics for Lithium Storage

Yuanhao Shen, Yalong Jiang, Zhongzhuo Yang, Jun Dong, Wei Yang, Qinyou An, and Liqiang Mai**

Y. H. Shen, Dr. Y. L. Jiang, Z. Z. Yang, W. Yang, Prof. Q. Y. An, Prof. L. Q. Mai
State Key Laboratory of Advanced Technology for Materials Synthesis and Processing, Wuhan University of Technology, Wuhan 430070, P. R. China

Prof. Q. Y. An, Prof. L. Q. Mai
Foshan Xianhu Laboratory of the Advanced Energy Science and Technology Guangdong Laboratory; Xianhu hydrogen Valley, Foshan 528200, China
E-mail: mlq518@whut.edu.cn (Prof. L. Q. Mai), anqinyou86@whut.edu.cn (Prof. Q.Y. An)

Dr. J. Dong
Hubei Engineering Research Center for Safety Monitoring of New Energy and Power Grid Equipment; Hubei University of Technology, Wuhan, 430068, P. R. China

Experimental Section

Materials synthesis:

Synthesis of α-MoO_3 nanobelts precursor. Firstly, 0.5 g molybdenum powder was put into the 50 ml beaker, and then slowly dropped 10 ml H_2O_2 into the beaker with a dropper. After stirring 30 min, the solution turned yellow and heat released. Subsequently, the homogeneous solution was transferred into a 50 ml Teflon-lined stainless-steel autoclave and heated at 200 °C for 24 h. After cooled down to room temperature naturally, the sample was washed by water and ethanol 3 times and dried at 60 °C under vacuum for 24 h.

Synthesis of mesoporous MoO_2 nanobelts (meso-MoO_2-NBs), mesoporous MoO_2/MoP heterostructure nanobelts (meso-MoO_2/MoP-NBs), mesoporous MoP nanobelts (meso-MoP-NBs).

meso-MoO_2-NBs: The α-MoO_3 precursor and sodium monophosphate hydrate ($NaH_2PO_2 \cdot H_2O$) (mass ratio 1: 4) were put into two boats separately and then annealed at 700 °C for 3 h under argon flow to gain meso-MoO_2-NBs.

meso-MoO_2/MoP-NBs: The α-MoO_3 precursor and sodium monophosphate hydrate ($NaH_2PO_2 \cdot H_2O$) (mass ratio 1: 8) were put into two boats separately and then annealed at 700 °C for 3 h under argon flow to gain meso-MoO_2/MoP-NBs.

meso-MoP-NBs: The α-MoO_3 precursor and sodium monophosphate hydrate ($NaH_2PO_2 \cdot H_2O$) (mass ratio 1: 16) were put into two boats separately and then annealed at 700 °C for 3 h under argon flow to gain meso-MoP-NBs.

The mass of α-MoO_3 precursor for meso-MoO_2-NBs, meso-MoO_2/MoP-NBs and meso-MoP-NBs is all 100 mg. The source and purity of all chemicals were listed in Table S1.

Materials characterization:

X-ray diffraction (XRD) patterns were collected by using a D8 Advance X-ray diffractometer with an area detector with Cu Kα radiation. The transmission electron microscopy (TEM) and scanning electron microscopy (SEM) images were taken on JEM-2100F STEM/EDS microscope and JEOL-7100F SEM microscope, respectively. Raman characterizations were measured with green laser (532 nm) using LABRAM HR Evolution Raman spectrometer. X-ray photoelectron spectroscopy (XPS) spectra were recorded using a VG MultiLab 2000 instrument. N_2 adsorption-desorption isotherms were measured by using a Tristar II 3020 instrument at liquid nitrogen temperature (77 K). The electrodes for ex-situ TEM experiments were prepared by taking apart the coin cells in an argon-filled glove box and the electrode materials were washed with alcohol and then dispersed in alcohol through ultrasonication.

Measurements of electrochemical performance:

The electrochemical properties were characterized by using 2016-type coin cells with lithium metal foil as the counter and reference electrode. The separator was Celgard. The electrode was all composed of 70 *wt*% active material, 25 *wt*% acetylene black and 5 *wt*% carboxymethyl cellulose (CMC) binder. The slurry was cast on Cu foil and dried in a vacuum oven at 60 °C for 12 h. The active material loading was 0.8-1 mg cm^{-2}. The 1.0 M $LiPF_6$ in EC: DMC: EMC=1:1:1 *v*ol% was used as electrolyte. The cells were assembled in an argon-filled glove box. Galvanostatic charge/discharge tests were undertaken on a multi-channel battery testing system (LAND CT2001A) with a cutoff voltage of 0.01-3 V *vs.* Li^+/Li. EIS and CV were measured on electrochemical workstation (Autolab PGSTAT302N). Mott–Schottky analysis was performed using the CHI 760E in a three-electrode cell. A glassy

graphite was used as the counter electrode in an aqueous 1 M KOH solution and Hg/HgO was used as reference electrode. All measurements were carried out at room temperature.

Density functional theory (DFT) calculations:

The present calculations were carried out by using the projector augmented wave (PAW)[1] method within the DFT as implemented in the Vienna ab initio simulation package (VASP).[2] The generalized gradient approximation (GGA) in the form of Perdew-Burke-Ernzerhof (PBE) was used to treat the exchange-correlation energy.[3] DFT-D3 scheme was applied to account for the van der Waals interactions during the calculations.[4] Usually, the cutoff energy for plane wave basis was based on the maximum cutoff (EMAX or ENMIN) value in the calculations with more than one species. We had conducted the cutoff energy test of 300, 350, 400, 450 and 500 eV to identify the appropriate cutoff energy. As shown in Figure S17 and Table S5, when the cutoff energy increases to 500 eV, the energy of MoP/MoO_2 system remains nearly unchanged. Thus, we choose the energy cutoff of 500 eV for wave functions expanded in plane wave basis. Slab models of 3×3×2 and 2×2×1 supercell with three atomic layers were constructed to simulate the (100) facet of MoP and the (101) facet of MoO_2. To demonstrate the effect of heterostructure, MoP (100) slab was added to the MoO_2 (101) slab to construct MoP (100)/MoO_2 (101) with a distance of about 2.2 Å. A vacuum slab of about 10 Å was inserted between the surface slabs for all the models. For the Brillouin-zone sampling, 2×2×2 of k-point was set for the structure relaxation and increased to 5×5×2 for the electronic structure calculations. Except for the bottom two atomic layers of the MoO_2 (101) slab and MoP (100) slab, all atoms were allowed to be fully relaxed while keeping the supercell boxes unchanged until the residual force per atom are less than 0.05 eV $Å^{-1}$. While

for MoP (100)/MoO_2 (101), the bottom two atomic layers of the MoO_2 (101) and top two atomic layers of the MoP (100) were fixed, other atoms were allowed to be fully relaxed while keeping the supercell boxes unchanged until the residual force per atom are less than 0.05 eV $Å^{-1}$. Ultrasoft pseudopotentials were used to describe the interaction of ionic core and valence electrons.

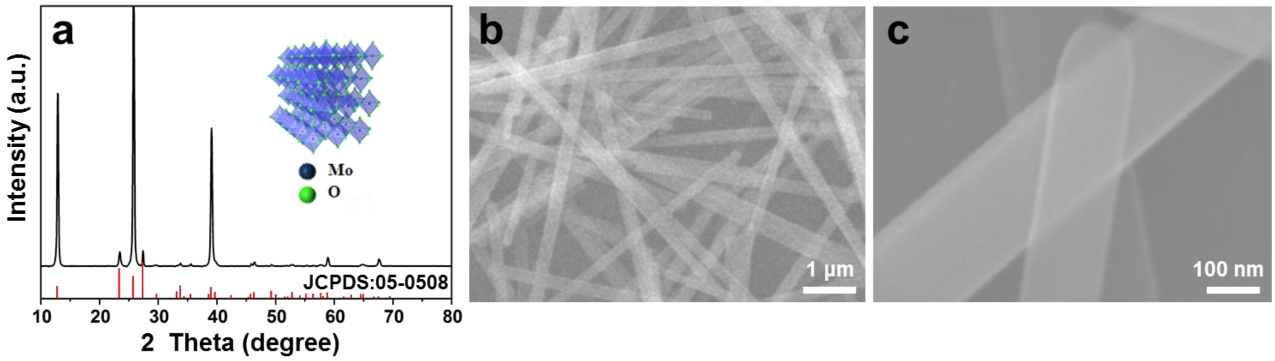

Figure S1. a) XRD pattern, b,c) SEM images of α-MoO_3 nanobelts.

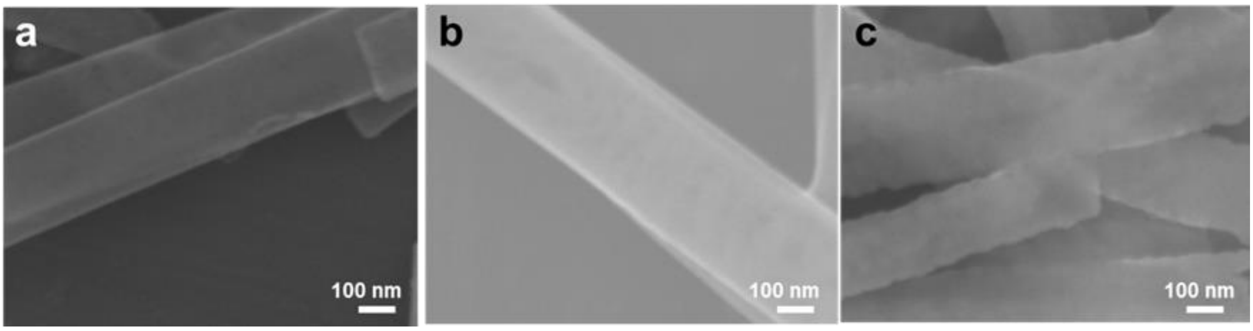

Figure S2. SEM images of a) meso-MoO_2/MoP-NBs, b) meso-MoO_2-NBs and c) meso-MoP-NBs, respectively.

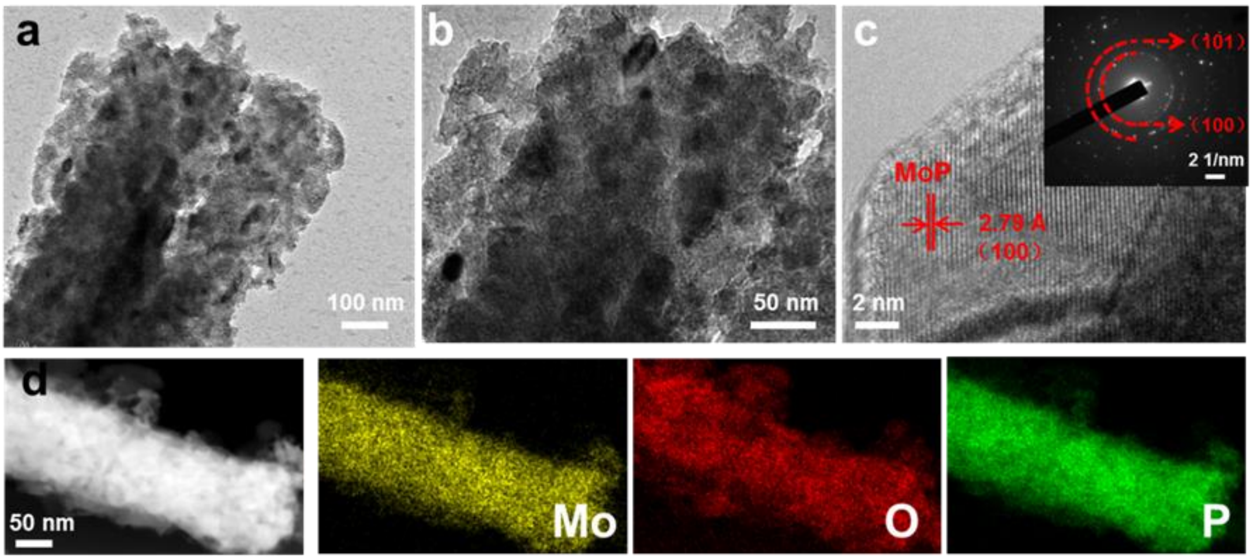

Figure S3. a,b) Bright field TEM images, c) HRTEM image and inset is the related SAED patterns, d) HAADF-STEM image and EDX mapping images of different elements of meso-MoP-NBs.

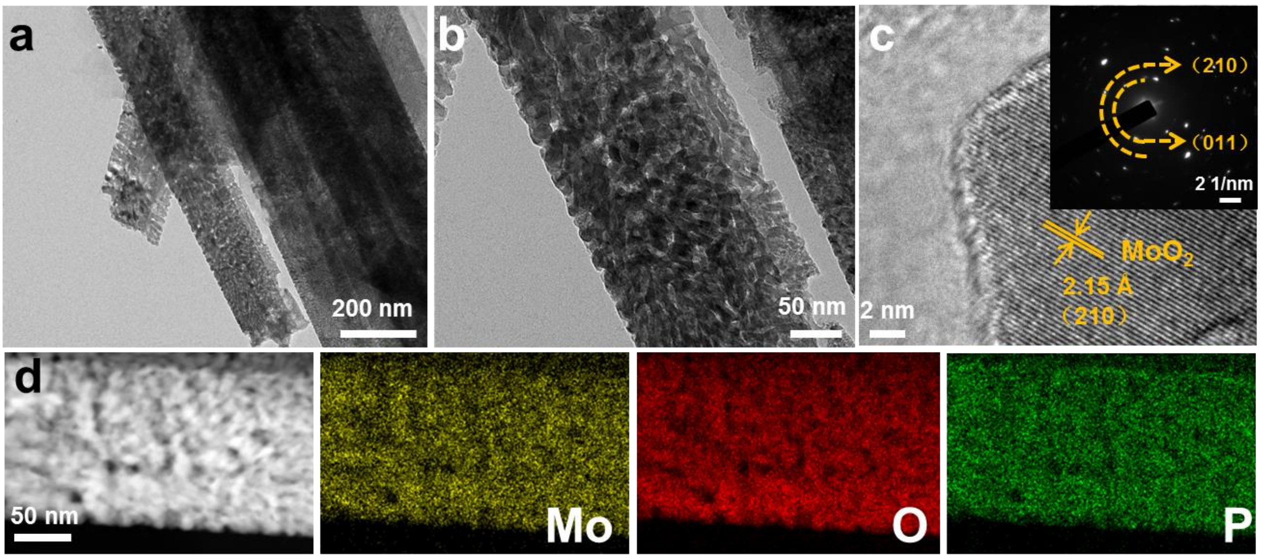

Figure S4. a,b) Bright field TEM images, c) HRTEM image and inset is the related SAED patterns, d) HAADF-STEM image and EDX mapping images of different elements of meso-MoO_2-NBs.

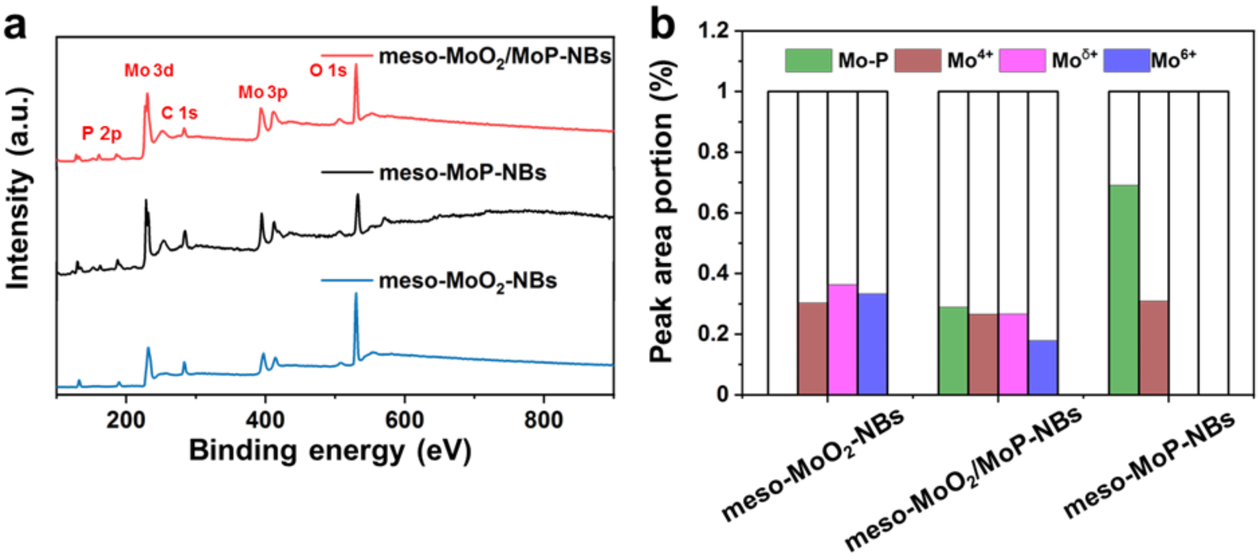

Figure S5. a) XPS survey spectra, b) The contribution of MoP and MoO_x (Mo^{6+}, $Mo^{\delta+}$and Mo^{4+}) calculated from XPS data for meso-MoO_2/MoP-NBs, meso-MoP-NBs and meso-MoO_2-NBs.

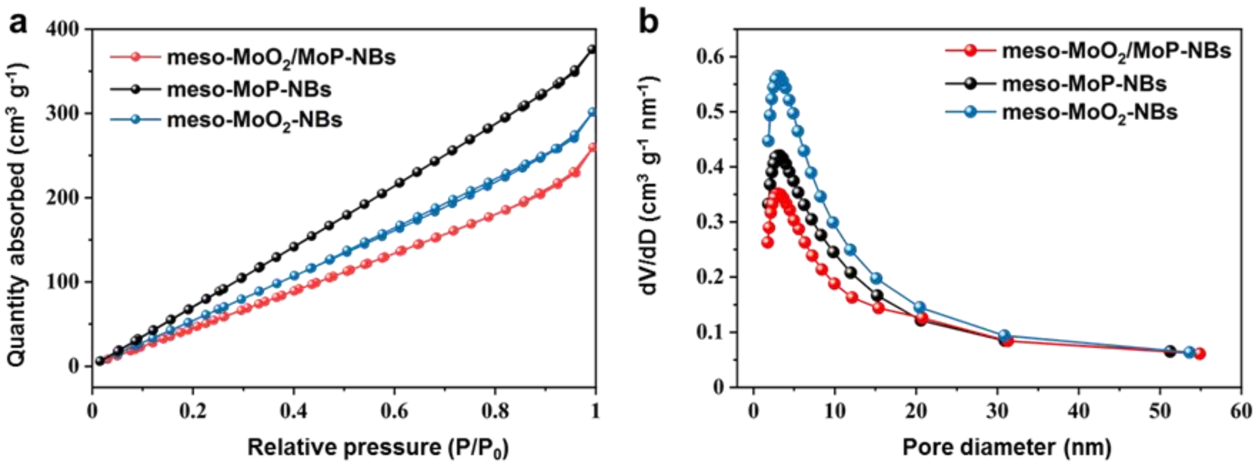

Figure S6. a) Nitrogen isotherm and b) Pore size distribution of meso-MoO_2/MoP-NBs, meso-MoP-NBs and meso-MoO_2-NBs.

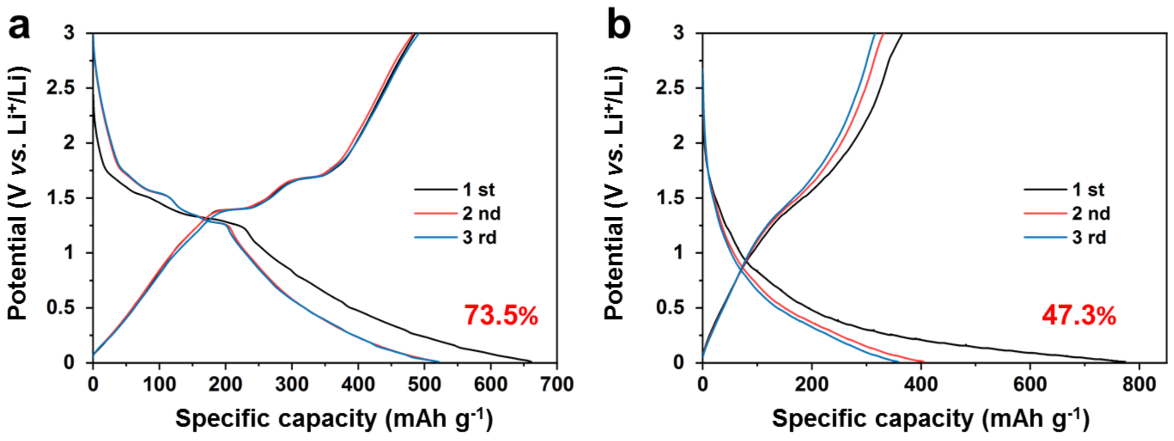

Figure S7. Galvanostatic charge–discharge curves at 0.1 A g^{-1} of a) meso-MoO_2-NBs and b) meso-MoP-NBs.

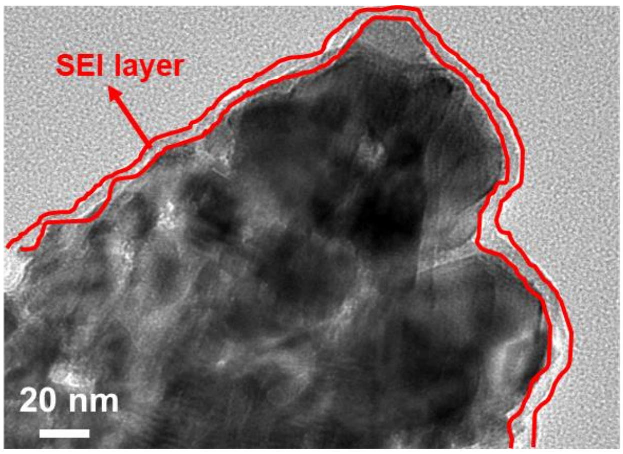

Figure S8. TEM image of meso-MoO_2/MoP-NBs after 5 cycles.

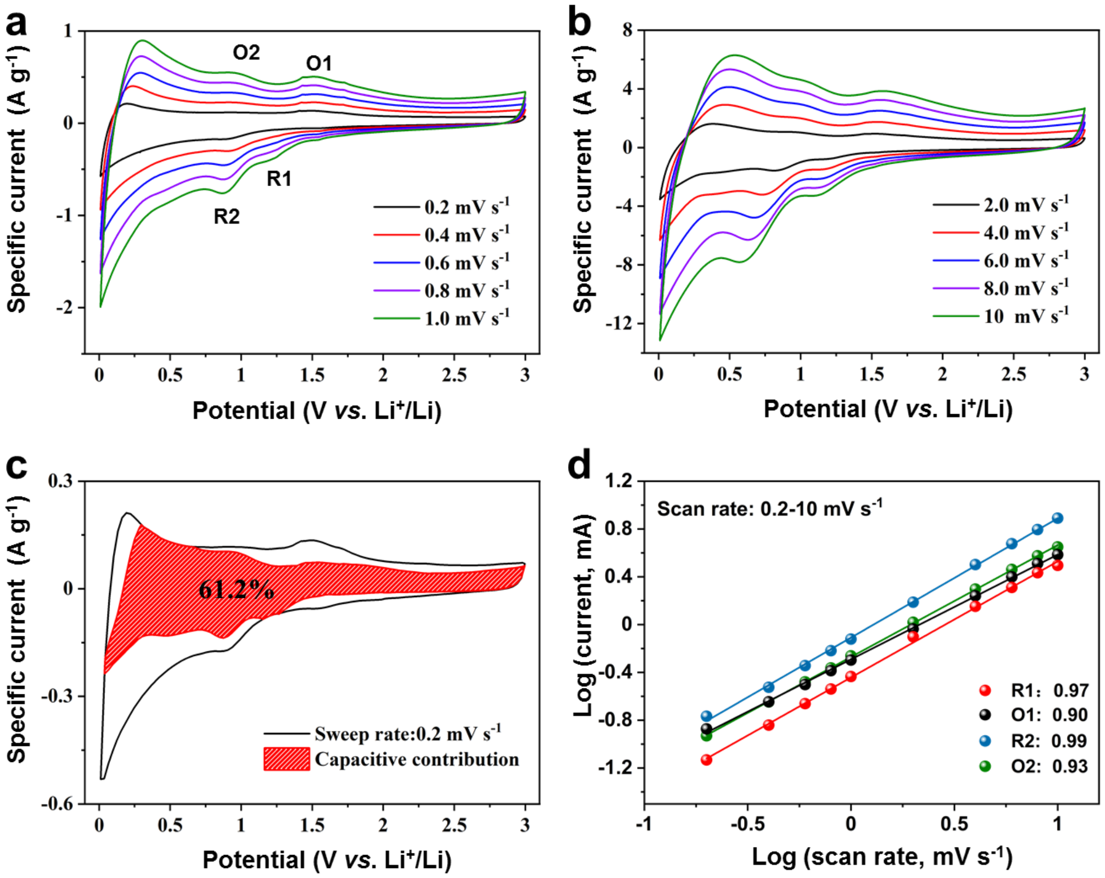

Figure S9. a,b) CV curves of meso-MoP-NBs at the sweep rates ranging from 0.2 to 10.0 mV s^{-1}. c) CV curve of meso-MoP-NBs at 0.2 mV s^{-1}, the hatched portion shows the capacitive controlled regions. d) Log(i) versus log(v) plots at different oxidation and reduction states of meso-MoP-NBs.

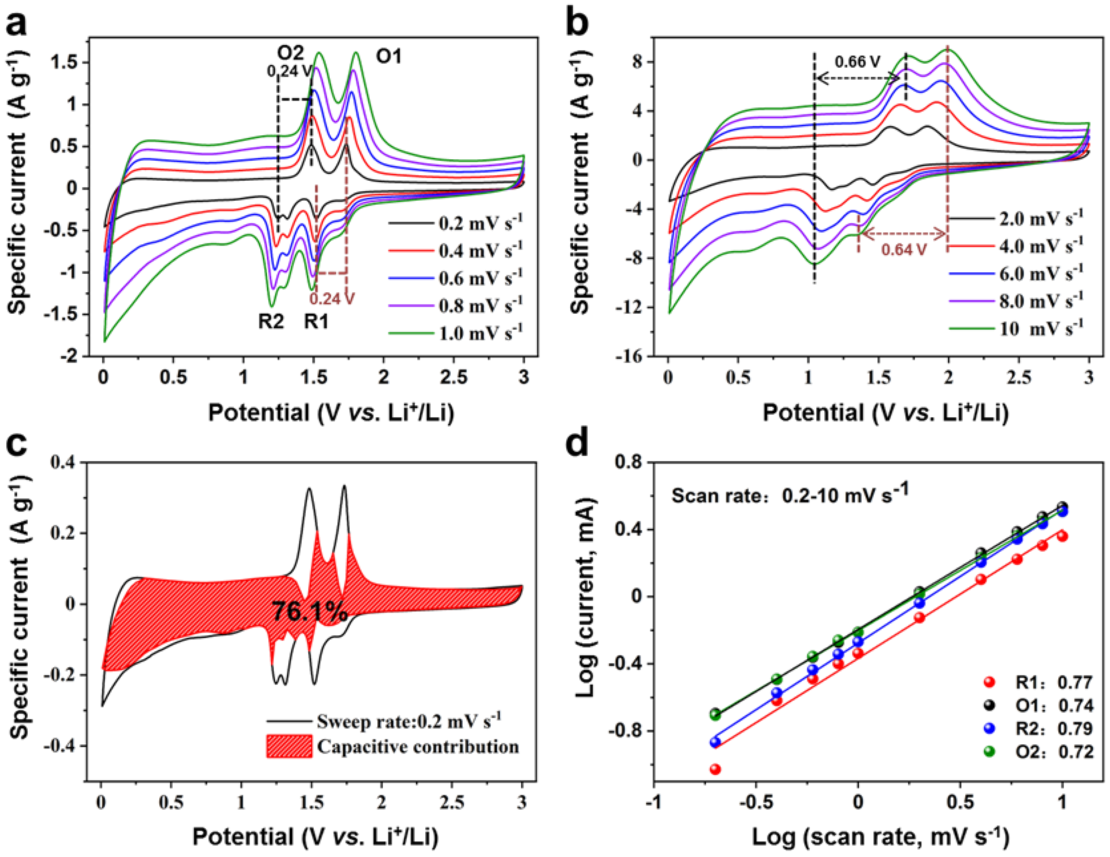

Figure S10. a,b) CV curves of meso-MoO_2-NBs at the sweep rates ranging from 0.2 to 10 mV s^{-1}. c) CV curve of meso-MoO_2-NBs at 0.2 mV s^{-1}, the hatched portion shows the capacitive controlled regions. d) Log (*i*) versus log (*v*) plots at different oxidation and reduction states of meso-MoO_2-NBs.

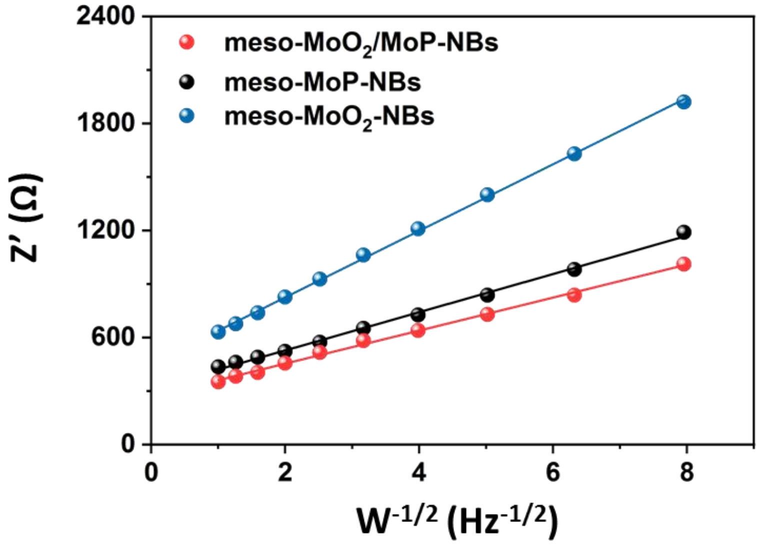

Figure S11. The kinetics calculations of meso-MoO_2/MoP-NBs, meso-MoP-NBs and meso-MoO_2-NBs based on the frequency (W) and Z' values at low frequency region.

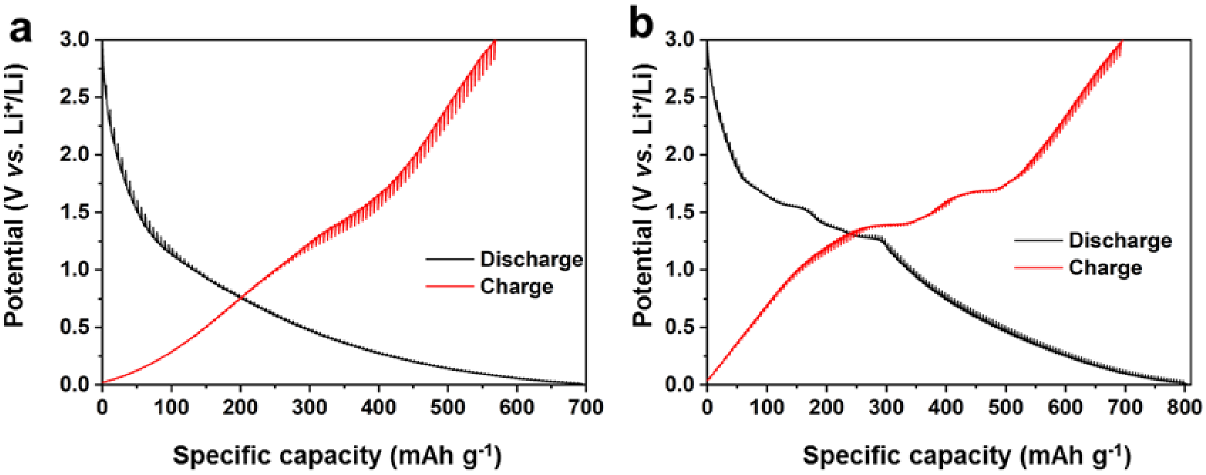

Figure S12. GITT curves of a) meso-MoP-NBs and b) meso-MoO_2-NBs, respectively.

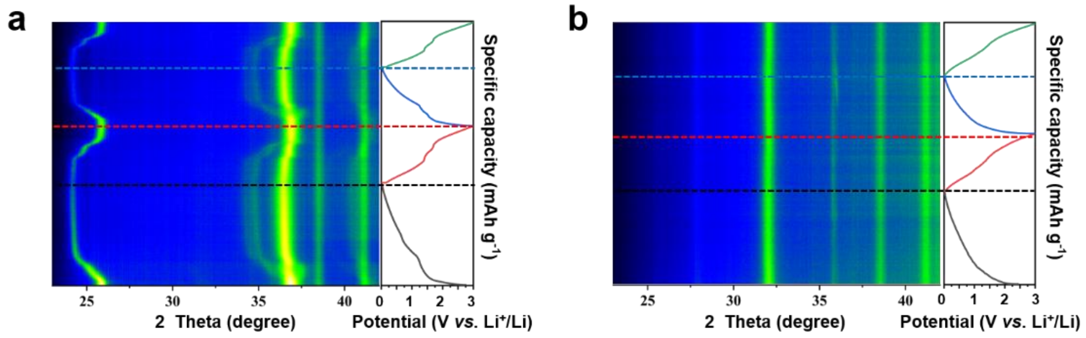

Figure S13. Operando XRD patterns of a) meso-MoO_2-NBs and b) meso-MoP-NBs at the first two cycles.

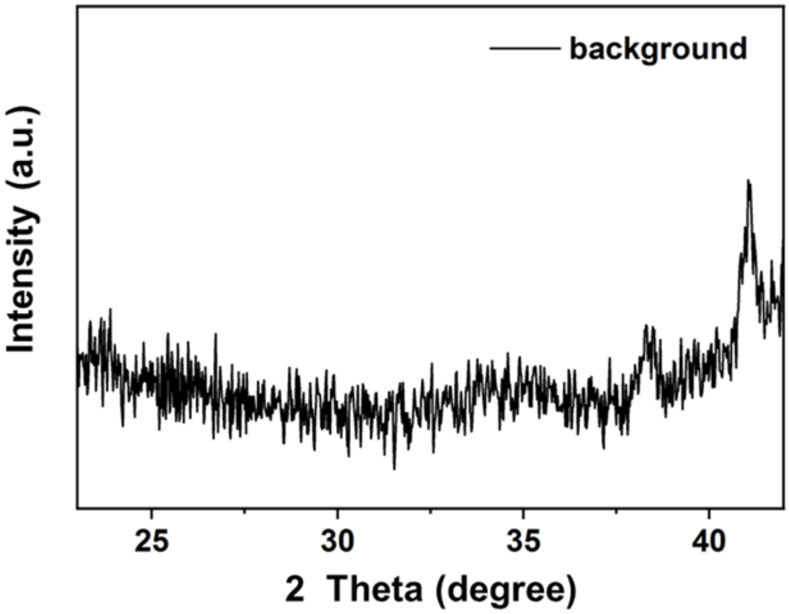

Figure S14. The basal peak of operando XRD.

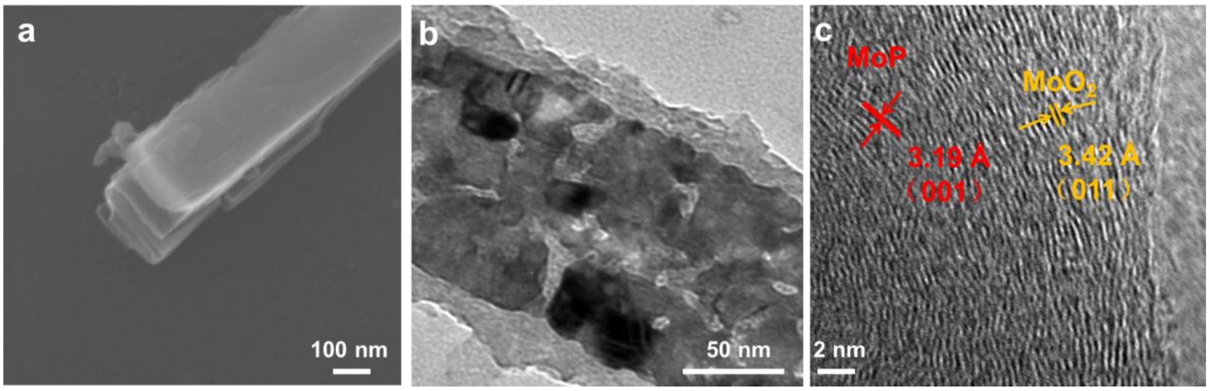

Figure S15. a) SEM images of meso-MoO_2/MoP-NBs after 50 cycles at 0.2 A g^{-1}. b) TEM and c) HRTEM images of meso-MoO_2/MoP-NBs after 1000 cycles at fully charged state.

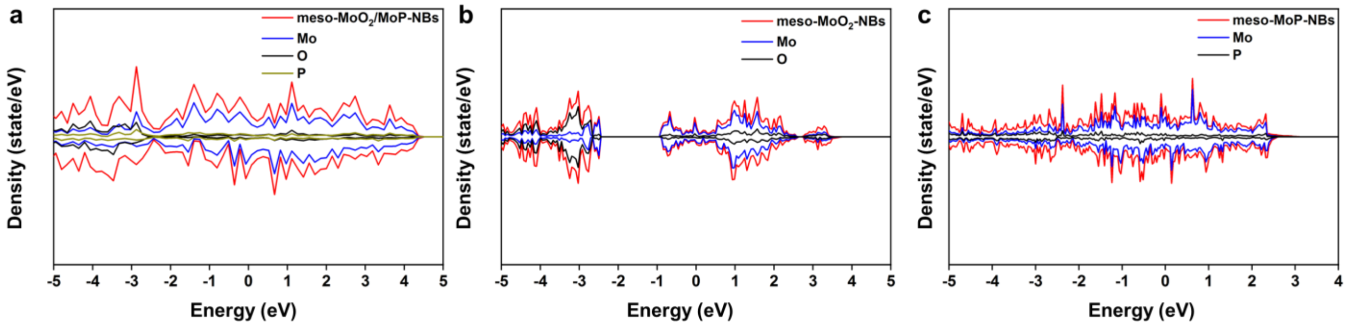

Figure S16. Partial density of states of a) meso-MoO_2/MoP-NBs, b) meso-MoO_2-NBs and c) meso-MoP-NBs.

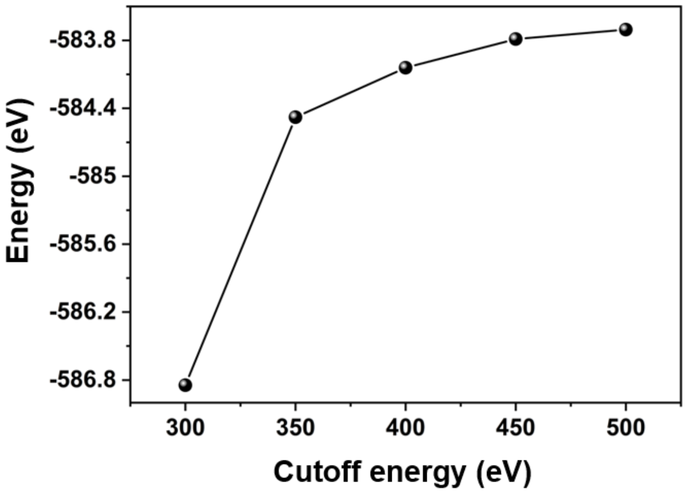

Figure S17. The test of cutoff energy for MoP/MoO_2 heterostructure.

Table S1. The source and purity of all chemicals.

Chemicals	Purity	Source
Mo powder	99.9%, metal basis <150 μm	Aladdin
H_2O_2	AR, ≥30%	Sinopharm Chemical Reagent Co. Lid., China
$NaH_2PO_2·H_2O$,	AR	Sinopharm Chemical Reagent Co. Lid., China

Table S2. ICP results of meso-MoO_2/MoP-NBs, meso-MoO_2-NBs and meso-MoP-NBs.

	Mo(wt.%)	P(wt.%)	O(wt.%)
meso-MoO_2/MoP-NBs	67.34	7.31	25.35
meso-MoO_2-NBs	64.79	6.14	29.07
meso-MoP-NBs	62.29	20.36	17.35

Table S3. The lattice parameter can be calculated by interplanar spacings of (011), (-211), (200) and (-212) planes and the formulae of lattice coefficient (a, b, c , h, k , l and β) in monoclinic system.

	a (Å)	b (Å)	c (Å)	V (Å^3)
initial state	5.6096	4.8570	5.6259	153.2822
discharge state (meso-MoO_2-NBs)	5.6576	4.9411	5.9591	166.5853
discharge state (meso-MoO_2/MoP-NBs)	5.6274	4.9332	5.9536	165.2784

Table S4. Comparison of the electrochemical performance of the meso-MoO_2/MoP-NBs with reported MoO_2-based anodes for LIBs.

Materials	Rate capability: Current density (A g^{-1})/capacity (mAh g^{-1})	Cycling performance: Current density (A g^{-1})/Cycle number/capacity (mAh g^{-1})	Ref.
MoO_2@C	5/146.1	0.5/200/1134	[5]
MoO_2 NP@rGO	2/673	1/350/641	[6]
MoO_2/C-G(M)	1/210	0.1/10/660	[7]
MoO_2/NC NPs	2/738	1/500/805	[8]
MoO_2/HCSs	0.4/774	0.5/100/1094	[9]
MoO_2@HPCNFs	2/425	0.1/100/1055	[10]
MoO_2/rGO	2/151	0.1/50/738	[11]
MoO_2@RGO	1.5/473	1/50/523	[12]
MoO_2@MoS_2	1/700	0.5/100/820.7	[13]
Mo_2N nanolayer coated MoO_2	5/415	0.1/100/815	[14]
**meso-MoO_2/MoP-NBs**	**8/291.2**	**1/1000/515**	**This work**

Table S5. The test of cutoff energy for MoP/MoO_2 heterostructure.

Cutoff energy (eV)	Energy of MoP/MoO_2 heterostructure (eV)
300	-586.846
350	-584.479
400	-584.042
450	-583.788
500	-583.705

Reference in Supporting Information

[1] G. Kresse, D. Joubert, *Phys. Rev. B* **1999**, 59, 1758.

[2] a) G. Kresse, J. Furthmüller, *Phys. Rev. B-Condens Matter* **1996**, 54, 11169; b) G. Kresse, J. Furthmüller, *Comput. Mater. Sci.* **1996**, 6, 15.

[3] J. P. Perdew, K. Burke, M. Ernzerhof, *Phys. Rev. Lett.* **1996**, 77, 3865.

[4] S. Grimme, S. Ehrlich, L. Goerigk, *J. Comput. Chem.* **2011**, 32, 1456.

[5] Y. Wang, H. Zhao, A. Di, X. Yang, B. Cong, G. Chen, *Int. J. Energy Res.* **2021**, 45, 9438.

[6] Y. Chu, B. Xi, S. Xiong, *Chin. Chem. Lett.* **2021**, 32, 1983.

[7] G. S. Zakharova, L. Singer, Z. A. Fattakhova, S. Wegener, E. Thauer, Q. Zhu, E. V. Shalaeva, R. Klingeler, *J. Alloy. Compd.* **2021**, 863, 158353.

[8] P. Zhang, S. Guo, J. Liu, C. Zhou, S. Li, Y. Yang, J. Wu, D. Yu, L. Chen, *J. Colloid Interface Sci.* **2020**, 563, 318.

[9] T. Rasheed, F. Nabeel, A. Naveed, S. Majeed, T. A. Sherazi, *Mater. Today Commun.* **2019**, 21, 100694.

[10] X. Chen, G. Gao, Z. Wu, J. Xiang, X. Li, G. Guan, K. Zhang, *RSC Adv.* **2019**, 9, 37556.

[11] S. Li, *Int. J. Electrochem. Sci.* **2018**, 13, 23.

[12] X. Chen, R. Liu, L. Zeng, X. Huang, Y. Fang, J. Liu, Y. Xu, Q. Chen, M. Wei, Q. Qian, *Mater. Lett.* **2017**, 212, 198.

[13] K. Zhu, J. Min, L. Yang, J. Luo, J. Liu, M. Lei, R. Zhang, L. Ren, Z. Wang, *Ionics* **2019**, 25, 1487.

[14] J. Liu, S. Tang, Y. Lu, G. Cai, S. Liang, W. Wang, X. Chen, *Energy Environ. Sci.* **2013**, 6, 2691.
